# Supplementary material for: Association Between Left Atrial Epicardial Adipose Tissue Attenuation Assessed by Cardiac Computed Tomography and Atrial Fibrillation Recurrence Following Catheter Ablation: A Systematic Review and Meta-Analysis
Source: J Clin Med. 2025 Jul 6;14(13):4771. doi: 10.3390/jcm14134771 (PMC12251091; doi:10.3390/jcm14134771)
Supplement: Supplementary file 1 [file jcm-14-04771-s001.zip › Table S2.pdf]

Table S2. Detailed Search Strategies Used in Each Database

Database	Search Strategy
PubMed	("atrial fibrillation"[MeSH Terms] OR "atrial fibrillation"[Title/Abstract]) AND ("epicardial adipose tissue"[Title/Abstract] OR "epicardial fat"[Title/Abstract] OR "epicardial adiposity"[Title/Abstract]) AND ("computed tomography"[MeSH Terms] OR "CT"[Title/Abstract]) AND ("attenuation"[Title/Abstract]) AND ("catheter ablation"[MeSH Terms] OR "ablation"[Title/Abstract])
Embase	('atrial fibrillation'/exp OR 'atrial fibrillation') AND ('epicardial adipose tissue':ti,ab OR 'epicardial fat':ti,ab) AND ('computed tomography'/exp OR 'CT':ti,ab) AND ('attenuation':ti,ab) AND ('catheter ablation'/exp OR 'ablation':ti,ab)
Cochrane Library	(atrial fibrillation):ti,ab,kw AND (epicardial adipose OR epicardial fat):ti,ab,kw AND (computed tomography OR CT):ti,ab,kw AND (attenuation):ti,ab,kw AND (ablation OR catheter ablation):ti,ab,kw
Web of Science	TS=("atrial fibrillation") AND TS=("epicardial adipose tissue" OR "epicardial fat") AND TS=("computed tomography" OR "CT") AND TS=("attenuation") AND TS=("ablation" OR "catheter ablation")
CINAHL	(MH "Atrial Fibrillation") AND ("epicardial adipose tissue" OR "epicardial fat") AND ("computed tomography" OR "CT") AND "attenuation" AND ("ablation" OR "catheter ablation")
