# Supplementary material for: Association Between Left Atrial Epicardial Adipose Tissue Attenuation Assessed by Cardiac Computed Tomography and Atrial Fibrillation Recurrence Following Catheter Ablation: A Systematic Review and Meta-Analysis
Source: J Clin Med. 2025 Jul 6;14(13):4771. doi: 10.3390/jcm14134771 (PMC12251091; doi:10.3390/jcm14134771)
Supplement: Supplementary file 1 [file jcm-14-04771-s001.zip › Table S3.pdf]

Table S3. Summary of computed tomography imaging details.

Author, Year	HU range	CT Scanner	Tube Voltage, kV
Beyer 2021	−195 to −5	single-source and dual-source >64-detector row scanners;	not defined
Hammache 2021	−250 to −50	256-slice multidetector cardiac scanner (Revolution CT, General Electric);	100
Li 2024	−190 to −30	third-generation dual-source CT device (Somatom FORCE; Siemens Healthineers) and 256-row CT device	not defined
Nodera 2024	−150 to −50	192-section dual-source SOMATOM Force CT scanner (Siemens Healthcare);	not defined
Yang 2022	−190 to −30	third-generation dual-source CT scanner (SOMATOM Force, Siemens Healthineers);	automatic tube-current
Ciuffo 2019	−200 to −50	320-detector CT scanner (Aquilion ONE; Toshiba Medical Systems);	80, 100, or 120 (depending on body habitus)
Mahdiui 2021	−195 to −45	256-slice CT scanner (Brilliance iCT 256, Phillips Healthcare);	100 to 120
